# Leukocyte-Derived Interleukin-10 Aggravates Postoperative Ileus

**DOI:** 10.3389/fimmu.2018.02599

**Published:** 2018-11-13

**Authors:** Kathy Stein, Mariola Lysson, Beatrix Schumak, Tim Vilz, Sabine Specht, Jürgen Heesemann, Axel Roers, Jörg C. Kalff, Sven Wehner

**Affiliations:** ^1^Department of Surgery, University of Bonn, Bonn, Germany; ^2^Institute for Medical Microbiology, Immunology and Parasitology, University of Bonn, Bonn, Germany; ^3^Department of Bacteriology, Max von Pettenkofer Institute, Munich, Germany; ^4^Institute for Immunology, Medical Faculty, Technical University Dresden, Dresden, Germany

**Keywords:** postoperative ileus, intestinal motility, interleukin-10, macrophages, neutrophils

## Abstract

**Objective:** Postoperative ileus (POI) is an inflammation-mediated complication of abdominal surgery, characterized by intestinal dysmotility and leukocyte infiltration into the muscularis externa (ME). Previous studies indicated that interleukin (IL)-10 is crucial for the resolution of a variety of inflammation-driven diseases. Herein, we investigated how IL-10 affects the postoperative ME inflammation and found an unforeseen role of IL-10 in POI.

**Design:** POI was induced by a standardized intestinal manipulation (IM) in C57BL/6 and multiple transgenic mouse strain including C-C motif chemokine receptor 2^−/−^, IL-10^−/−^, and LysM^cre^/IL-10^fl/fl^ mice. Leukocyte infiltration, gene and protein expression of cytokines, chemokines, and macrophage differentiation markers as well as intestinal motility were analyzed. IL-10 serum levels in surgical patients were determined by ELISA.

**Results:** IL-10 serum levels were increased in patient after abdominal surgery. In mice, a complete or leucocyte-restricted IL-10 deficiency ameliorated POI and reduced the postoperative ME neutrophil infiltration. Infiltrating monocytes were identified as main IL-10 producers and undergo IL-10-dependent M2 polarization. Interestingly, M2 polarization is not crucial to POI development as abrogation of monocyte infiltration did not prevent POI due to a compensation of the IL-10 loss by resident macrophages and neutrophils. Organ culture studies demonstrated that IL-10 deficiency impeded neutrophil migration toward the surgically traumatized ME. This mechanism is mediated by reduction of neutrophil attracting chemokines.

**Conclusion:** Monocyte-derived macrophages are the major IL-10 source during POI. An IL-10 deficiency decreases the postoperative expression of neutrophil-recruiting chemokines, consequently reduces the neutrophil extravasation into the postsurgical bowel wall, and finally protects mice from POI.

## Introduction

Postoperative ileus (POI) is a common sequela of abdominal surgery and is characterized by gastrointestinal dysmotility. POI is associated with increased morbidity and prolonged hospitalization, subsequently leading to a significant medico-economic burden (1). The intensity of the operative trauma was shown to correlate with the duration of POI in rodents and humans (2–4). Previous work of our group and others demonstrated that POI originates from an inflammation of the muscularis externa (ME) that is triggered by activation of resident macrophages (5) and enteric glia (6). The inflammatory course can be separated into an early and a late phase. The early manifestation phase involves the local production of pro-inflammatory mediators, including interleukin (IL)-1α/β, IL-6, tumor necrosis factor alpha (TNF-α), monocyte chemokine ligand (CCL)-2, or kinetic active mediators like nitric oxide that all aggravate the inflammation and abrogate smooth muscle contractility (7, 8). This phase is followed by a cell infiltration, particularly by blood-derived monocytes and neutrophils (2, 9–11). Therefore, modulation of the underlying immune response to the surgical trauma has become an important target opening new avenues for preventive or therapeutic measures (12). While POI in general is a self-limiting disease, patients often suffer from prolonged POI which majorly affects patient's recovery after abdominal surgery. Although the mechanisms of prolonged POI are unknown, it may depend on an insufficient counter-regulation that may lead to an overwhelming and prolonged inflammation. Recent studies suggest that pro-resolving lipid mediators are involved (13) in the resolution of POI but also anti-inflammatory cytokines have been hypothesized to be involved (14).

Inflammation resolving mechanisms were often described to involve M2 macrophage polarization which substantially differ from the proinflammatory M1 phenotype (15, 16). M2 macrophages promote resolution of inflammation and wound healing and express higher levels of Arginase 1 (Arg1), transforming growth factor (TGF)-β, and IL-10 (17, 18). Prominent mediators that drive M2 polarization are IL-4 (15) and IL-10 (19–21). A previous study has shown that IL-10 recombinant IL-10 reduced POI in mice (14) but the mechanism behind remained elusive. Herein, we hypothesized that IL-10 may affect POI by triggering M2 macrophage. Indeed we found that IL-10 plays a crucial role in M2 macrophage polarization but strikingly, we identified an unforeseen role of IL-10 in different transgenic mouse lines which challenges our pervious knowledge about its role during POI development. Mechanistically, this role depends on an alteration of neutrophil recruitment to the surgically manipulated site of the bowel.

## Materials and Methods

### Animals

Experiments were performed with WT 8- to 12-week-old male C57BL6/J mice (Janvier, Saint Berthevin Cedex, France) with a mean body weight of 20–25 g. Additionally, CCR2^−/−^, ITIB^+/−^ (22), IL10^−/−^, IL4^−/−^LysM^cre^/IL10^flfl^, and LysM^cre^-TdTomatoSTOP^fl/fl^ mice were used. CCR2-, IL-10-, and IL-4-deficient mice were obtained from Jackson Laboratories (Charles River, Sulzfeld, Germany). LysM^cre^-TdTomatoSTOP^fl/fl^ mice were kindly provided by Prof. Irmgard Förster (LIMES Institute, University of Bonn). All experiments were performed in accordance to federal law for animal protection and approved by proper authorities of North-Rhine-Westfalia (LANUV), file numbers 87-51.04.2010-A091, 87-51.04.2010-A284, 84-02.04.2013.A162, and 84-02.04.2013.A104.

### Patients

IL-10 serum concentrations were quantified by an IL-10 Elisa (R&D Systems, Wiesbaden, Germany) in 24 patients of which 17 underwent open abdominal and 7 underwent extraabdominal surgery. Blood samples were taken before and 24 h after surgery. Patients were recruited from the University Hospital Bonn during the BiPOI trial which was registered at the German Clinical Trial Register, file DRKS00000736. The study protocol (#065/11) was approved by the ethical committee of the University of Bonn and all patients gave written informed consent in accordance with the Declaration of Helsinki.

### Operative Procedures

The small intestine of mice was mechanically manipulated in a standardized manner as described previously (2). Briefly, after laparotomy the small bowel was eventrated and manipulated by two moisted cotton tips from oral to aboral direction. After intestinal manipulation (IM), the intestine was placed back and the abdomen closed by a two layer suture. Control animals did not undergo IM.

### Flow Cytometry Analyses

Small bowel ME immunocytes were isolated by enzymatically digestion of the ME with 0.1% Collagenase type II (Worthington, New York, USA) diluted in HBSS containing 0.1 mg/mL DNase I (La Roche, Mannheim, Germany), 2.4 mg/mL Dispase II (La Roche), 1 mg/mL BSA (Applichem, Darmstadt, Germany), and 0.7 mg/mL Trypsin inhibitor (Applichem) for 35 min in a 37°C shaking water bath. Afterwards the cell suspension was filtered through 70 μm gauze and single cells were analyzed by flow cytometry. ITIB^+/−^ mice express beta-lactamase together with IL-10 under control of the endogenous IL-10 promotor. Cells from ITIB^+/−^ and control littermate mice were resuspended in a CCF4-AM staining solution (CCF4, Life Technologies, Darmstadt, Germany), according to the manufacturer's instructions before, washed with FACS buffer (PBS, 1% FBS, and 2 mM EDTA) and subjected to immunofluorescence staining. Fluorescence resonance energy transfer of CCF4-AM was used to visualize IL-10 production at single-cell level (Supplementary Figure 1A). For all cell analyses, Fc-blocking solution (clone 2.4G2) was incubated for 20 min and cells further stained for 30 min at 4°C with fluorochrome-labeled monoclonal antibodies against CD45, F4/80, Ly6C, and Ly6G (see Supplementary Table 2). Gating strategies included exclusion of dead cells [Hoechst-33342^+^ (Sigma)], Zombie NIR™ solution or Propidium Iodide, respectively, doublet and CD45^−^ cells. Additional gating strategies are mentioned in the figure legends. Flow cytometry analyses were performed on a FACS Canto II (BD Biosystems, Heidelberg, Germany) and data analyzed with FlowJo software (Tree Star Inc., Ashland, USA).

### Cell Sorting

For each cell sort, ME tissue of 12 mice was pooled. The extraction of ME immunocytes was done as described above. Bone marrow derived monocytes and neutrophils were isolated by flushing the bone marrow cells out of femur and tibia with ice-cold PBS by using a 25-gauge needle. In another approach, immunocytes from the ME of 2–3 WT or IL10^−/−^ naïve mice were isolated like described above and CD45^+^F4/80^+^ resident macrophages were sorted. Samples were acquired and sorted by using FACS Diva software (BD Biosciences, Heidelberg, Germany) on FACS Aria flow cytometer (BD Biosciences, Heidelberg, Germany).

### Arginase Activity

Arginase activity was evaluated in mouse ME tissue as described before (23). Results were specified as urea produced per hour per 100 mg tissue.

### Tissue Culture and Determination of IL-10 Release in Supernatant

POI was induced in WT, CCR2^−/−^, IL4^−/−^, and IL10^−/−^ and LysM^cre^IL10^fl/fl^ mice and the ME harvested 24 h after manipulation. The isolated muscularis (50 mg) was incubated (37°C, 5% CO_2_) in 6-well tissue culture plates within 500 μL of DMEM. After 24 h, cell-free supernatant was collected and stored at −80°C. IL-10 release from these ME organ cultures was measured by a commercial ELISA (BD Biosciences, Heidelberg, Germany). The given results were normalized to pg/100 mg ME tissue.

### IL-10 Reconstitution in LysM^cre^IL10^fl/fl^ Mice

POI was induced in 8–10 weeks old LysM^cre^IL10^fl/fl^ mice and rmIL-10 (1,000 ng/250 μL sterile PBS, Biolegend, Germany) was injected intraperitoneally 3 h after IM. Twenty-four hours after manipulation MPO^+^ cells, GIT, and cell infiltration were determined.

### Neutrophil Transmigration Assay

Bone marrow neutrophils were isolated from naïve WT mice as described before (24). 10^5^ neutrophils were resuspended in 100 μL RPMI 1% FCS and placed into the upper chamber of a Transwell insert (6.5 mm, 3 μm pore, polycarbonate membrane, Corning Incorporated, New York, USA) in triplicates. The lower chamber contained RPMI 1% FCS supplemented with 20% of cell-free supernatant gained from 24 h ME incubation of unmanipulated WT or IL-10^−/−^ mice (10 mg ME/100 μL RPMI), respectively. In a second approach the lower chamber contained RPMI 1% FCS supplemented with 10 or 100 ng/mL rmIL-10 (ImmunoTools, Germany). The cells were incubated for 3 h at 37°C in 5% CO_2_. Cells, which migrated from the upper to the lower chamber through the filter, were collected and counted by a hemocytometer.

### Measurement of Gastrointestinal Transit (GIT)

GIT was measured 24 h after IM. Animals were gavaged with a FITC-labeled dextran solution (size 70 kDa; Sigma-Aldrich, Steinheim, Germany) 22.5 h after IM. Ninety minutes later, the complete GI tract was harvested and divided into 15 segments (stomach–colon) and FITC dextran contents were fluorometrically determined in each segment. The geometric center (GC) was calculated as described previously by distribution of the FITC-labeled dextran using this formula: GC = ∑ (% of total fluorescent signal per segment ^*^ segment number)/100.

### Quantitative RT-PCR

Total RNA of ME tissue samples were extracted using Trizol (Life Technologies, Darmstadt, Germany) according to manufacturer's instructions. Sorted cells were extracted using PicoPure Isolation RNA kit (Thermo Fischer Scientific, Germany). Complementary DNA was synthesized using the High Capacity cDNA Reverse Transcription Kit (Applied Biosystems, Darmstadt, Germany). Expression of mRNA was quantified in triplicates by a RT-PCR with exon-spanning primers, Quantitect primer assay (Qiagen), or TaqMan probes (Supplementary Table 1) and SYBR Green PCR Master Mix or the TaqMan Gene Expression Master Mix (Applied Biosystems) were used for the PCR.

### Histochemical and Immunofluorescence Analysis

Midjejunal, ME whole mounts were fixed in ethanol for 10 min and underwent an staining of myeloperoxidase (MPO)+ cells for 10 min with 1 mg/ml Hanker Yates reagent (Polysciences Europe, Hamburg, Germany) in PBS containing 0.3% H_2_O_2_. MPO^+^ cells were counted in five randomly chosen areas and calculated as MPO^+^ cells/mm^2^.

In some experiments ME whole mounts from C57BL6/L or LysM^cre^-TdTomatoSTOP^fl/fl^ underwent 4% paraformaldehyde fixation for 30 min, were blocked with 3% bovine serum albumin in PBS, permeabilized by 0.2% Triton-X 100, and were incubated with either anti-MHC-II or anti-F4/80 or anti-Arg1 antibodies overnight followed (Supplementary Table 2) by incubation with FITC- or Cy3-coupled secondary IgG antibodies (Dianova, Hamburg, Germany, 1:1,600 diluted). Confocal laser scanning microscopy (Zeiss, LSM 700, Oberkochen, Germany) was used for analysis. MPO and MHCII analyses in LysM^cre^-TdTomatoSTOP^fl/fl^ mice were performed with a TE2000 microscope (Nikon, Düsseldorf, Germany).

### Statistical Analysis

Statistical analysis was performed with Prism V5.04 (GraphPad, San Diego, CA) using unpaired Student's *t*-test or one-way or multivariate ANOVA as indicated followed by Bonferroni's *post-hoc* test. Levels of significance were indicated as *p* < 0.05 (^*^), *p* < 0.01(^**^), and *p* < 0.01(^*^). The statistical methods used in each analyses are mentioned in the figure legends.

## Results

### Leukocyte Restricted IL-10 Deficiency Limits Inflammation and Neutrophil Extravasation in POI

IL-10 is described as a cytokine with anti-inflammatory properties. In the present manuscript we describe an unforeseen detrimental role of IL-10 in the pathogenesis of POI.

**Figure 1:**
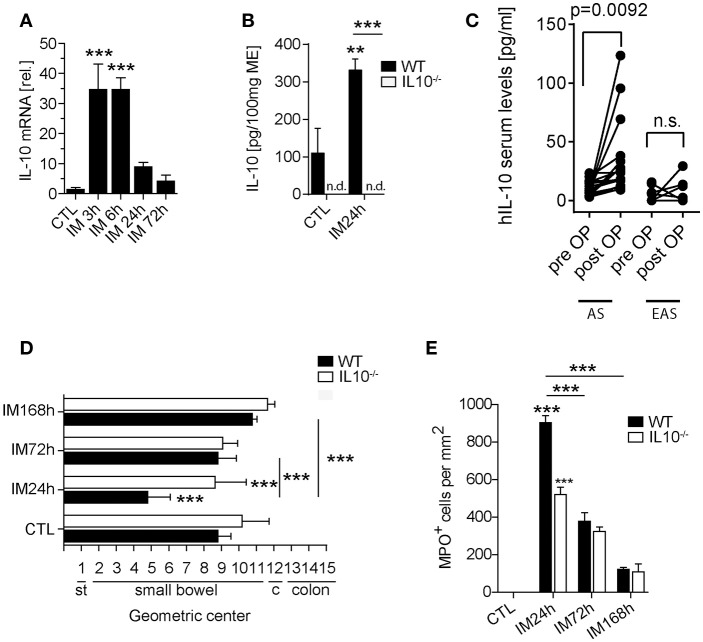
The role of IL-10 in POI. WT and IL-10^−/−^ mice underwent IM or left untreated (CTL). **(A)** Gene expression of IL-10 was analyzed in WT mice after indicated time points and compared to naïve controls (CTL). *n* = 5 mice per group. Statistical analysis was done by one-way ANOVA followed by Bonferroni's *post-hoc* test (***p < 0.001). **(B)** IL-10 release into the media of 24 h organ cultured ME harvested from intestinal manipulated (IM24h) WT or IL-10^−/−^ mice. Groups were compared by one-way ANOVA followed by Bonferroni's *post-hoc* test (**p < 0.01 and ***p < 0.001 vs. control or indicated groups; n.d. not detected). Bar graphs demonstrate means ± SEM, *n* = 4 for both groups. **(C)** IL-10 human serum concentrations were measured in surgical patients who underwent abdominal (AS, *n* = 17) and extraabdominal (EAS, *n* = 7) surgery before and 24 h after surgery. Data sets are shown as means ± SEM and were compared via a paired *t*-test. **(D)** GI-transit was plotted as mean ± SEM calculated by the geometric center of distribution of a fluid meal. st, stomach, c, cecum. Bar graphs show means ± SEM and are representative for five independent experiments. **(E)** MPO+ cells were quantified in naïve and 24, 72, and 168 h after IM in WT and IL-10^−/−^ mice. Cells were microscopically counted in five randomly chosen areas in ME whole mount specimens. Statistical analysis was performed by one-way ANOVA followed by Bonferroni's *post-hoc* test (***p < 0.001 vs. CTL or indicated groups).

**Figure 2:**
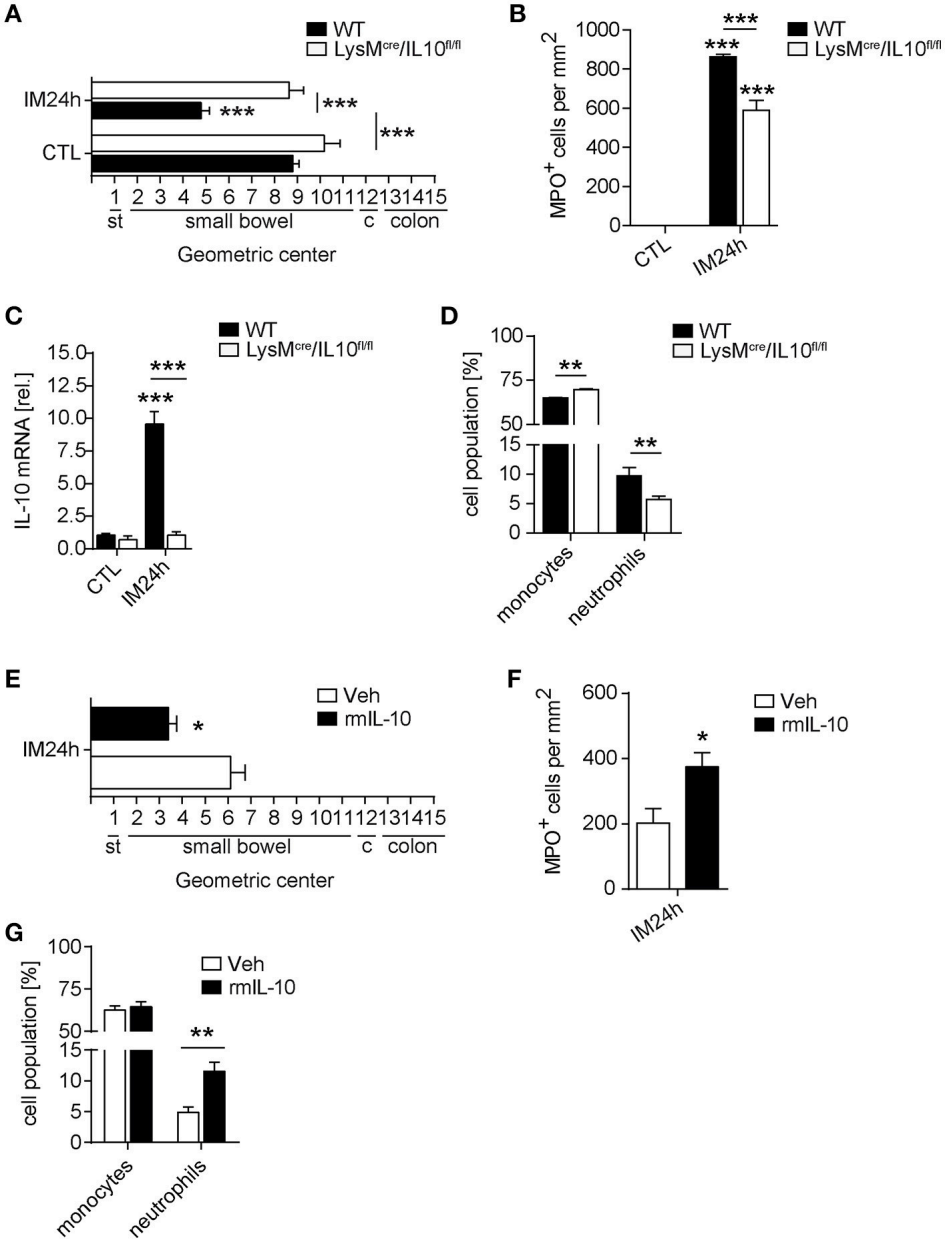
Leukocyte restricted IL-10 deficiency limits inflammation and neutrophil extravasation in POI. WT and LysM^cre^/IL10^fl/fl^ mice underwent IM. **(A)** GI-transit was plotted as mean ± SEM calculated by the geometric centers of distribution of a fluorescent marker. st, stomach, c, cecum. Bar graphs show means ± SEM and are representative for five independent experiments. **(B)** Quantification of MPO^+^ cells within the same animals used in **(A)**. Statistical analysis was performed with a one-way ANOVA followed by Bonferroni's *post-hoc* test (***p < 0.001 vs. CTL or indicated groups). **(C)** Gene expression analysis of IL-10 was performed 24 h after IM and compared to naïve controls (CTL). Data are shown as mean fold change ± SEM in mRNA levels vs. CTL. *n* = 5 for all groups. ***p < 0.001 vs. strain-specific CTL or between indicated samples. Statistical analysis was done by one-way ANOVA followed by Bonferroni's *post-hoc* test. **(D)** Flow cytometry analysis of ME specimen was performed to identify Ly6G^−^Ly6C^+^ monocytes and Ly6G^+^Ly6C^+^ neutrophils 24 h after IM in WT and LysM^cre^/IL10^fl/fl^ mice. Data sets are shown as mean fold change ± SEM, *n* = 5 for all groups. Statistical significance between mouse strains was determined by unpaired *t*-test (**p < 0.01 vs. indicated samples). **(E–G)** LysM^cre^/IL10^fl/fl^ mice were treated with rmIL-10 or vehicle after IM. GI-transit **(D)**, MPO^+^ cells **(E)**, and flow cytometry analysis **(F)** of ME specimen was performed as described above. Data sets (*n* = 4) were compared by unpaired *t*-test (*p < 0.05, **p < 0.01 vs. vehicle treated group).

In order to analyze whether IL-10 is involved in POI we first analyzed its gene expression in a time course of POI. After IM, IL-10 was more than 35-fold upregulated in the ME (Figure 1A) compared to control mice. IL-10 protein was also increased in supernatants of postoperative ME organ cultures (Figure 1B) taken from animals 24 h after IM while IL-10 was absent in IL-10^−/−^ mice, confirming the correct genotype of these mice and specificity of the ELISA. In patients that underwent open abdominal surgery, we also observed an increase in postoperative IL-10 serum levels (Figure 1C) while patients with extraabdominal surgery did not exhibit elevated IL-10 levels. From these findings, we hypothesized that IL-10 may be involved in POI pathophysiology and resolution and determined GI transit time and leukocyte extravasation in IL-10^−/−^mice. Unexpectedly, we observed a significantly improved GI-transit 24 h after surgery in IL-10^−/−^mice compared to WT animals. No differences in transit time were observed at day 3 and 7 in IL-10^−/−^mice (Figure 1D). The functional data were supported by reduced numbers of MPO^+^ leukocytes 24 h after IM (Figure 1E), which majorly consist of Ly6C^+^Ly6G^−^ monocytes (84%) and Ly6C^+^Ly6G^+^ neutrophils (13%) (Supplementary Figure 1A). Interestingly, these findings are contradictory to previous data that described delayed recovery from POI and even death at day 7 after IM (14). As huge variety of cell types is able to produce IL-10 we subjected mice with a leukocyte-restricted IL-10 deficiency to IM. Conditional targeting was achieved by use of LysM-cre recombinase mice (25). A pilot experiment revealed LysM expression in MHC-II^+^ resident muscularis macrophages in naïve LysM^cre^TdTomatoSTOP^fl/fl^ mice (Supplementary Figure 1B). After IM, LysM-cre expression was detected in almost all living CD45^+^ infiltrating Ly6C^+^ monocytes also Ly6G^+^ neutrophils and more than 60% of the resident F4/80^+^Ly6C^−^ macrophages (Supplementary Figure 1B). Given that LysM-cre mice indeed exhibit cre-recombinase activity in leukocytes, we next crossbred them to IL-10^fl/fl^ and subjected the LysM^cre^IL10^fl/fl^ littermates to our POI model. Remarkably, LysM^cre^IL10^fl/fl^ mice showed a similar outcome as pan-IL-10 deficient mice with a normalized GI-transit (GC: 8.6 ± 1.8) 24 h after IM compared to WT mice (Figure 2A) and significantly reduced MPO^+^ leukocyte counts (Figure 2B). As expected, the IM-induced increase in IL-10 gene expression was not observed in LysM^cre^IL10^fl/fl^ mice (Figure 2C). Further characterization of the LysM^cre^IL10^fl/fl^ inflammatory infiltrate revealed a more than 40% reduction in Ly6C^+^Ly6G^+^ neutrophils compared to WT mice (Figure 2D). Simultaneously, Ly6C^+^Ly6G^−^ monocytes were slightly increased in LysM^cre^IL10^fl/fl^ mice.

As IL-10 deficiency attenuated POI in two independent mouse strains we concluded that IL-10 deficiency protects mice from POI. In turn, an application of exogenous IL-10 to these mice should restore the POI phenotype observed in WT mice and impair the postoperative outcome of IL-10 deficient mice. Indeed, recombinant IL-10 administration reversed the improved POI phenotype of LysM^cre^IL10^fl/fl^ mice which developed a regular POI as shown by a decelerated GI transit time and increased MPO^+^ leukocyte influx (Figures 2E,F). In line with the reduction of neutrophils in LysM^cre^IL10^fl/fl^, recombinant IL-10 administration increased the number of neutrophils but not monocytes (Figure 2G).

### IL-10 Is Mainly Produced by Monocytes and Macrophages During POI

**Figure 3:**
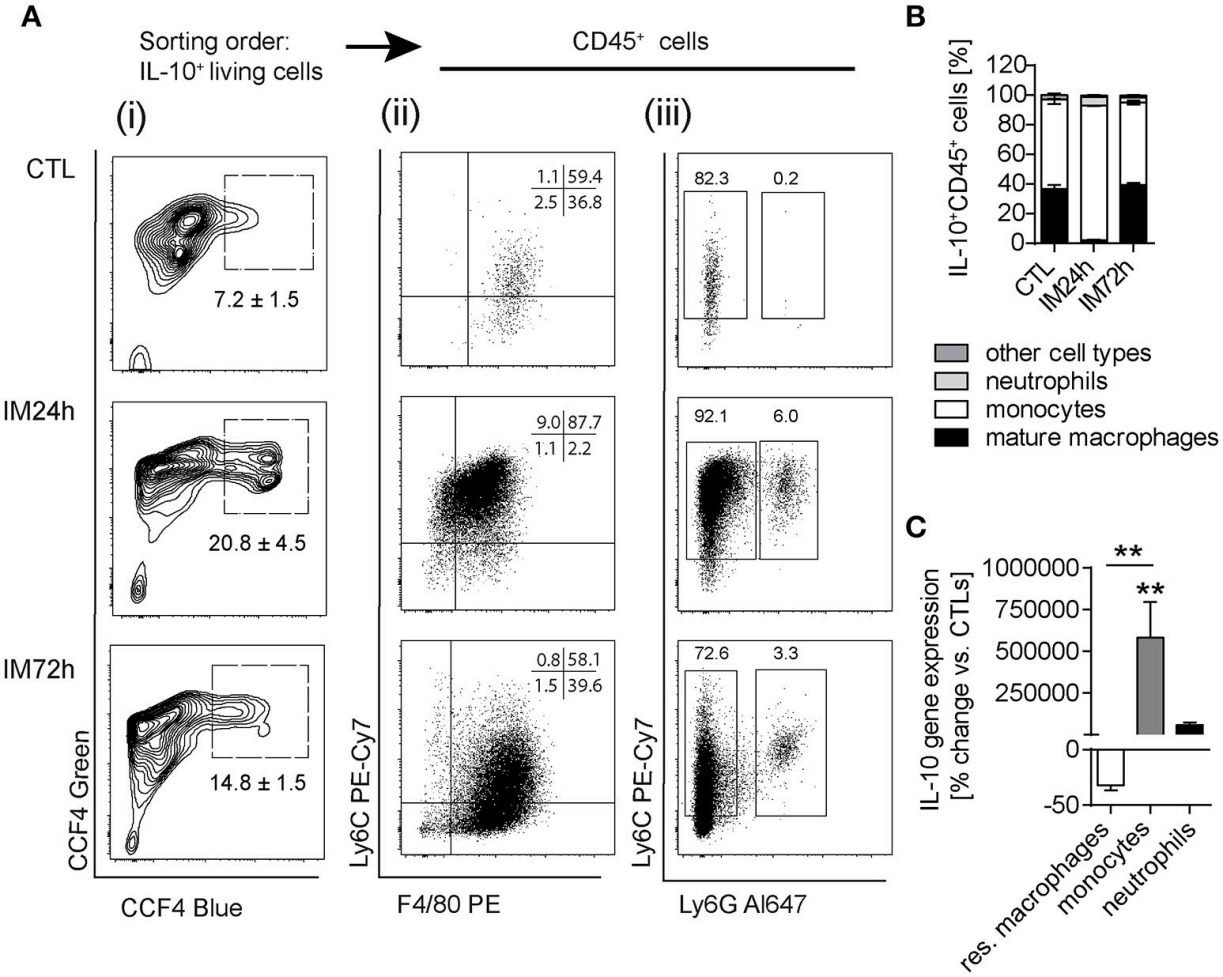
IL-10 is mainly produced by monocytes and macrophages during POI. **(A)** ITIB^+/−^ mice underwent IM or left untreated (CTL). After 24 and 72 h, IL-10 reporter activity was detected by a CCF4 administration resulting in blue fluorescence. Gating on living IL-10^+^ cells **(i)** was followed by CD45+ as well as identification of F4/80^+^/Ly6C^+/−^ **(ii)** and Ly6C^+^Ly6G^+/−^ **(iii)** cell populations during flow cytometry analysis. Data are representative plots from three independent experiments. **(B)** Ratios of IL-10^+^CD45^+^ cells within the ME of CTL and intestinally manipulated WT mice. Data are shown as means ± SEM, *n* = 3 for all groups. **(C)** Resident F4/80^+^Ly6C^−^ macrophages, Ly6C^+^Ly6G^−^ monocytes, and Ly6C^+^Ly6G^+^ neutrophils were sorted by flow cytometry from CTL and IM24 h animals, respectively. Indicated cell populations underwent gene expression analysis of IL10. *n* = 3 for all groups. Statistical analysis was done by one-way ANOVA followed by Bonferroni's *post-hoc* test. **p < 0.01 vs. CTL.

We next aimed to specify the cellular source of IL-10 by use of ITIB mice, a sensitive IL-10 reporter mouse strain (Supplementary Figure 2A) (22). In naïve ITIB^+/−^ mice, IL-10 was expressed within 7.2% ± 1.5 of the ME cells (Figures 3Ai,B) of which almost all were CD45^+^ leukocytes (98.97% ± 0.6). Of those, 36.8% ± 4.6 were F4/80^+^Ly6C^−^ resident macrophages and 59.4% ± 5.6 Ly6C^+^ Ly6G^−^ monocytes (Figure 3Aii). Ly6C^+^Ly6G^+^ neutrophils were absent in naïve mice (Figure 3Aiii). The total number of IL-10^+^ cells was increased at 24 h (20.8% ± 4.5) and 72 h (14.8% ± 1.5) after surgery (Figure 3Ai). Although F4/80^+^Ly6C^−^ macrophages were among IL-10^+^ cells (2.2% ± 0.5), infiltrating monocytes were the predominant IL-10 expressing cell population 24 h after IM (92.1% ± 0.9, Figure 3Aiii,3B). After 72 h, the F4/80^+^Ly6C^−^IL-10^+^ mature macrophage population consisting of resident ME macrophages and macrophages descendants of the Ly6C^+^ monocytes, increased (39.6% ± 2.2). Ly6G^+^IL-10^+^ neutrophils increased to 6.0% ± 0.9 but decreased at 72 h (3.3% ± 0.1, Figure 3Aiii,B). Additionally, gene expression analyses of postoperatively sorted monocytes, neutrophils and macrophages confirmed abundant IL-10 gene expression in infiltrated monocytes compared to bone marrow-derived monocytes. This upregulation was not observed in sorted ME neutrophils (compared to bone marrow derived neutrophils) nor in resident ME macrophages (Figure 3C). Taken together, these data show that infiltrating F4/80^+^Ly6C^+^ monocytes are the predominant source of IL-10 during POI.

Next we were wondering if an elimination of this IL-10 source would also affect POI. Recently, monocyte infiltration during POI was shown to depend on CCR2 (26, 27). Consistent with these data, F4/80^+^Ly6C^+^ cells were almost absent in CCR2^−/−^ (1.8% ± 0.8) compared to WT mice (62% ± 1.7) 24 h after IM (Supplementary Figure 3A) while F4/80^+^Ly6C^−^ resident macrophages were not affected by CCR2 deficiency (Supplementary Figure 3B). Surprisingly, CCR2^−/−^ and WT mice demonstrated the same extent of motility disturbances 24 h after IM (GC: 4.1 ± 0.6 and 4.1 ± 0.4, respectively, Supplementary Figure 3C). Surprisingly, postoperative ME IL-10 protein levels were not reduced but even increased in CCR2^−/−^ mice (Supplementary Figure 3D), indicating that compensatory mechanisms encounter the IL-10 loss in monocytes. A comparison of CCR2-deficient ITIB^+/−^ and CCR-2 competent ITIB^+/−^ mice demonstrated that the numbers of IL-10 producing resident macrophages and neutrophils increased, indicating that these cells compensate the IL-10 loss in monocytes (Supplementary Figure 3E).

### M2 Polarization Is Driven by IL-10 but Does Not Affect POI

**Figure 4:**
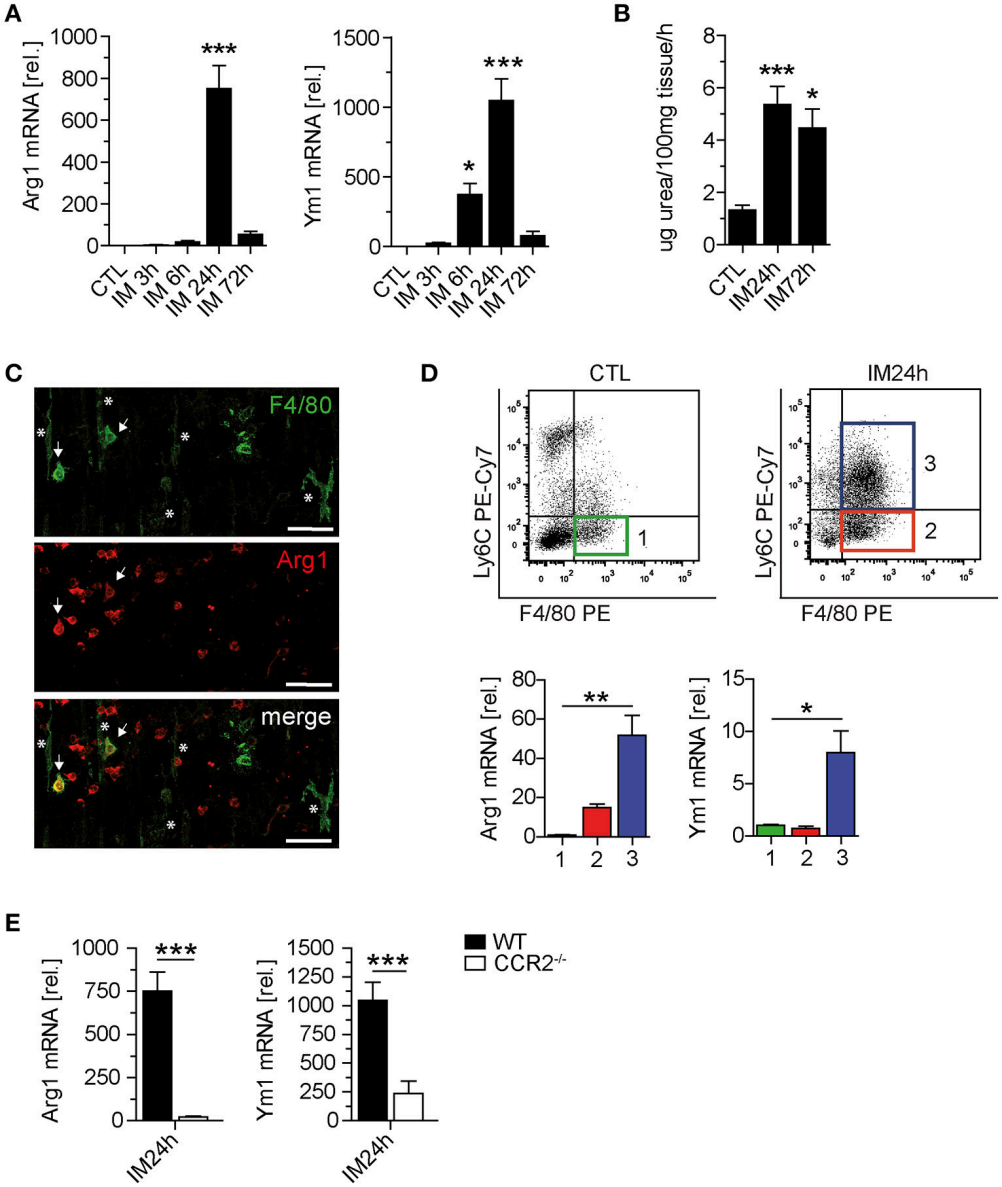
IL-10 is mainly produced by monocytes and macrophages during POI. **(A)** Arginase 1 (Arg1) and Ym1 mRNA levels were analyzed in WT mice after indicated time points. *n* = 5 mice per group. **(B)** Arginase activity was determined by urea formation within the same ME specimens used in **(A)**. **(C)** Confocal microscopy of F4/80 and Arg1 expressing cells 24 h after IM in the ME of WT mice. Asterisks, resident F4/80^+^/Arg1^−^ muscularis macrophages; arrows, infiltrated F4/80^+^Arg1^+^ monocytes. Note the presence of numerous Arg1^+^ monocyte-shaped cells not yet expressing F4/80. Shown are representative images from three independent experiments. Scale = 50 μm. **(D)** Three different cell populations (1–3) were sorted by flow cytometry from CTL and IM24h animals. Populations were defined as F4/80^+^Ly6C^−^ resident macrophages (1, CTL green; 2, IM24h red) and F4/80^+^Ly6C^+^ monocytes (3, IM24h blue). Indicated cell populations underwent gene expression analysis of Arg1 and Ym1. *n* = 3 for all groups. Statistical analysis was done by one-way ANOVA followed by Bonferroni's *post-hoc* test. *p < 0.05 and **p < 0.01 vs. group 1. **(E)** Gene expression of Arg1 and Ym1 was analyzed 24 h postoperatively in WT and CCR2^−/−^ mice. Data are shown as changes in mRNA levels (means ± SEM) compared to non-manipulated strain specific controls. Groups (*n* = 5) were compared via unpaired *t*-test (***p < 0.001).

IL-10 triggers M2 polarization of macrophages. Therefore, we analyzed if M2 polarization occurs and affects POI. In WT mice, Arg1 and Ym1 mRNA expression was increased and peaked 24 h after IM (Figure 4A). Accordingly, the ME arginase activity was also increased up to 72 h after IM (Figure 4B). To characterize the cells undergoing M2 polarization during POI, we microscopically identified numerous F4/80^+^ round-shaped monocytes in the postoperative ME (Figure 4C). Arg1 immunoreactivity was rarely found in F4/80^+^ stellate-shaped resident macrophages. Quantitative PCR of sorted ME leukocytes confirmed elevated Arg1 expression in infiltrating F4/80^+^Ly6C^+^ monocyte-derived macrophages (Figure 4D, group 3) compared to F4/80^+^Ly6C^−^ resident macrophages from naïve (group 1) and intestinally manipulated mice (group 2). Arg-1 expression did not differ between group 1 and 2. In line with these findings, CCR2^−/−^ mice, lacking F4/80^+^Ly6C^+^ monocyte-derived macrophages, as well as IL-10^−/−^ and LysM^cre^IL10^fl/fl^ mice demonstrated strongly reduced postoperative Arg1 and Ym1 mRNA levels compared to WT mice (Figure 4E). Furthermore, ME macrophages of naïve IL-10^−/−^ mice exhibited a pro-inflammatory gene expression profile in comparison to WT mice (Supplementary Figure 4). Notably, we did not detect any signs of ME leukocyte infiltration in naïve ME (Figure 1E), confirming that IL-10 deficient mice, known to spontaneously develop colitis at a certain age, exhibit no basal inflammation in the ME. We subsume that IL-10 triggers M2 polarization of naïve resident macrophages and infiltrating monocyte-derived macrophages during POI.

Finally we analyzed of IL-13 or IL-4, two other prototypical M2 polarization-triggering cytokines may also be involved in POI. IL-13 transcripts were only slightly elevated (~3-fold) in the early phase of POI (Supplementary Figure 4B) and IL-4 was not detected in the naïve or postoperative ME but in the spleen (Supplementary Figure 4B). However, a previous study detected ME IL-4 transcripts (27). To ensure that we did not overlook IL-4-driven effects we subjected IL-4^−/−^ mice to IM. Neither Arg1 activity nor MPO^+^ leukocyte counts or GI transit differed between WT and IL-4^−/−^ mice (Supplementary Figures 4C–E), indicating that IL-4 is not involved in M2 polarization during POI. Mice with a deficiency in IL13 signaling were not available to us.

### Neutrophil Transmigration Is Impaired by IL-10 Deficiency

**Figure 5:**
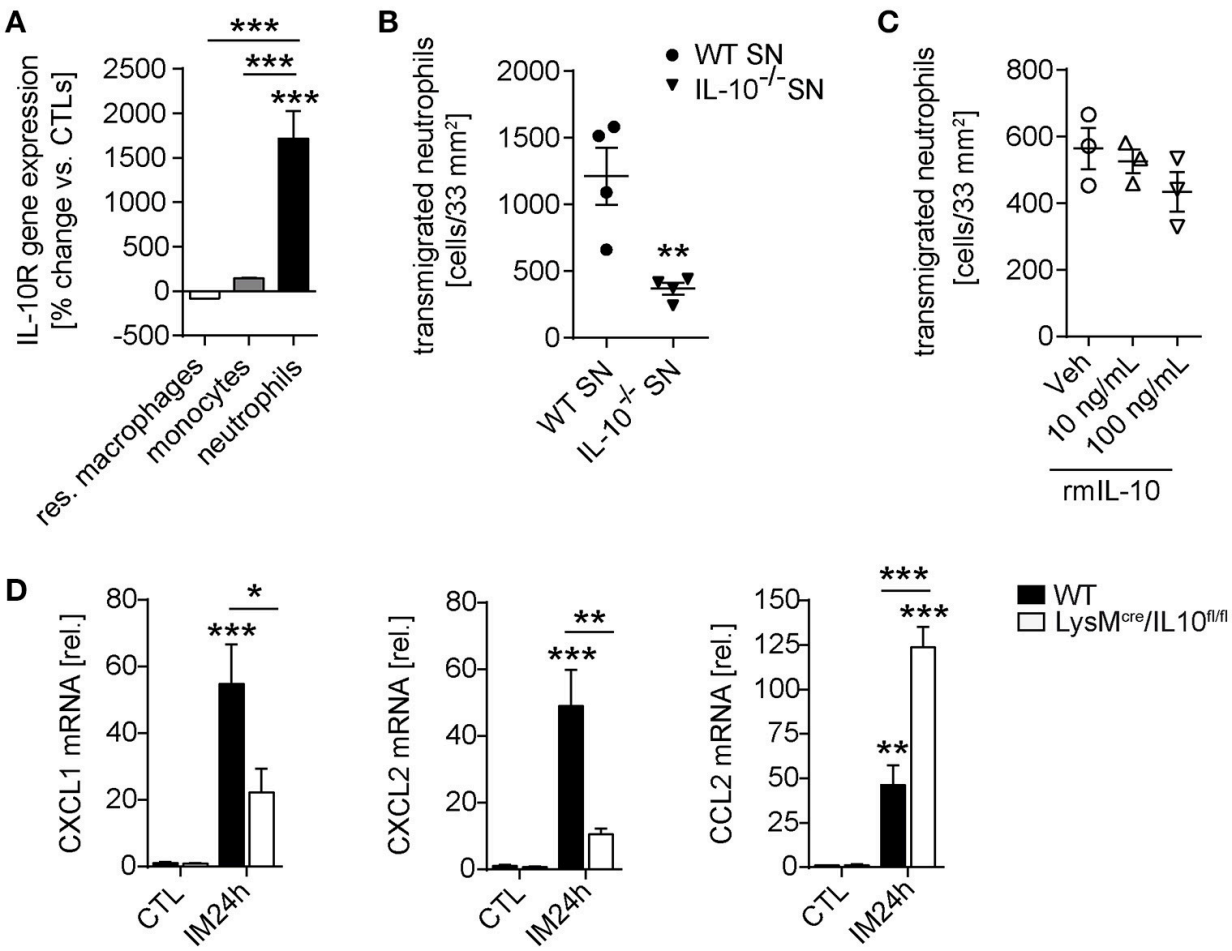
Neutrophil transmigration is impaired by IL-10 deficiency. **(A)** Resident F4/80^+^Ly6C^−^ macrophages, Ly6C^+^Ly6G^−^ monocytes and Ly6C^+^Ly6G^+^ neutrophils were sorted by flow cytometry from CTL and IM24h animals, respectively. Indicated cell populations underwent gene expression analysis of IL-10Rα. *n* = 3 for all groups. Statistical analysis was done by one-way ANOVA followed by Bonferroni's *post-hoc* test. ***p < 0.001 vs. CTL or indicated populations. **(B)** Neutrophil transmigration was determined toward WT ME or IL-10^−/−^ ME conditioned media (SN, supernatant). Data are shown as mean fold change ± SEM. *n* = 4 for both groups. Statistical significance was determined by unpaired *t*-test (**p < 0.01 vs. WT SN). **(C)** Analysis of neutrophil transmigration through a microporous membrane. Bone marrow derived WT neutrophils were cultured in triplicates in a transwell culture system and where were allowed to transmigrate toward IL-10 supplemented media (10, 100 ng/mL) or medium (Veh). No significance was determined between vehicle and IL-10 supplemented groups. **(D)** WT and LysM^cre^/IL10^fl/fl^ mice underwent IM and qPCR analysis of neutrophil chemokines CXCL1/CXCL2 as well as monocyte chemokine CCL2 was performed 24 h after IM and compared to naïve controls (CTL). Data are shown as mean fold change ± SEM in mRNA levels vs. CTL. *n* = 5 for all groups. *p < 0.05; **p < 0.01; ***p < 0.001 vs. strain-specific CTL or between indicated samples. Statistical analysis was done by one-way ANOVA followed by Bonferroni's *post-hoc* test.

In order to further identify the mechanism by which IL-10 deficiency ameliorates POI we spend attention on the significantly reduced neutrophil numbers in IL-10^−/−^ and LysM^cre^IL10^fl/fl^ mice. Postoperatively, we sorted extravasated neutrophils, monocytes and resident ME macrophages and neutrophils demonstrated a more than 1,700-fold increased IL-10Rα gene expression compared to naïve bone marrow neutrophils. The infiltrated monocytes and resident macrophages did not upregulate IL-10Rα compared to bone marrow-derived monocytes or resident ME macrophages from naïve animals (Figure 5A). Interestingly, we also observed a reduced transmigration of WT neutrophils to the site of traumatized ME organ culture supernatants from IL-10 deficient mice compared to WT mice (Figure 5B). We hypothesized that IL-10 may directly affect neutrophil migration and compared toward recombinant IL-10 directly. Neither 10 nor 100 ng/ml IL-10 altered the number of migrating neutrophils (Figure 5C), indicating that IL-10 indirectly affects neutrophil. As neutrophil recruitment requires chemotaxis we quantified mRNA of prototypical neutrophil attracting chemokines in the ME. In contrast to WT mice, the neutrophil attracting chemokines CXCL1 and CXCL2 decreased 24 h after IM in LysM^cre^IL10^fl/fl^ mice, whereas CCL2 increased under IL-10 deficiency (Figure 5D). These findings suggest that IL-10 affects neutrophil migration to traumatized sites via regulation of neutrophil chemokine expression.

Overall our results identified a so unforeseen role for IL-10 in POI's pathogenesis wherein IL-10 triggers neutrophil extravasation to the surgically manipulated bowel and ameliorates POI.

## Discussion

Resolution of POI is crucial for the recovery of patients undergoing abdominal surgery. Leukocyte recruitment into the intestinal ME has been shown to be a hallmark of POI in rodents, swine, and humans (5, 27). Another study demonstrated that IL-10 is involved in the resolution of POI (6) and we hypothesized that the infiltrating leukocytes could be a major source or target of IL-10 and might induce and/or undergo M2 polarization which contributes to healing processes and finally resolution of POI.

A time course of IL-10 gene expression indicated that IL-10 is upregulated within the first 24 h and might play a role in the early phase. This finding is in line with a previous study of 10 patients, demonstrating an early increase of IL-10 within 4 h after major abdominal (28). Our present results demonstrate that serum levels from patient undergoing open abdominal but not from patients with extraabdominal surgery are still significantly increased 24 h after surgery. Of note, the abdominal surgery group showed a significantly higher recovery time from POI than the extraabdominal surgery group (29). This indicates that IL-10 might modulate the postoperative immune response during, particularly in the first 24 h. However, a previous study demonstrated no effect of IL-10 deficiency 24 h after surgery but observed an impairment in POI resolution at later time points and even observed death of IL-10^−/−^ in the postoperative phase (6). Therefore, we repeated this experiment and subjected IL-10^−/−^ mice to IM. Surprisingly, we found that IL-10^−/−^ mice did not exhibit a delayed resolution but rather demonstrated an accelerated GI transit. Beneficial effects of an IL-10 deficiency have also observed in a model of surgical skin wounding skin (30). The sharp difference between these previous findings by Stoffels et al. and our data prompted us to subject LysM^cre^IL10^fl/fl^ mice to IM and they exhibited exactly the same phenotype like the IL-10^−/−^ animals did in our hands. The LysM-driven restriction of IL-10 deficiency was chosen as monocytes, monocyte-derived macrophages, and neutrophils are the predominant infiltrating cell types indicating that they might be the major IL-10 source or target in POI. LysM^cre^IL10^fl/fl^ mice (25) have been shown to be deficient of IL-10 in leukocytes in naïve mice and other inflammatory disease models (31–33). By use of LysM^cre^-TdTomatoSTOP^fl/fl^ mice we confirmed leukocyte restriction to infiltrating leucocytes and resident macrophages of the ME. The striking differences to Stoffels's et al. findings may be explained by technical reasons. While we used a single intraperitoneal injection of 40 μg/kg BW 3 h after IM, Stoffels et al. used two subcutaneous injections, 1 h prior and 1 h after IM, each with 125 μg/kg BW of recombinant IL-10. Another reason for this divergence could be the different application time points of IL-10. Previous studies suggested that IL-10-mediated effects are time dependent as IL-10 neutralization at the time of a cecal ligation and puncture (CLP)-induced sepsis increased the sepsis-induced mortality (34). However, lethality decreased when IL-10 was neutralized after CLP (35). These studies confirm the divergent data regarding the protective or harmful roles of IL-10. However, as we observed the same phenotype in two independent IL-10 deficient mouse models (IL-10^−/−^ and LysM^cre^IL10^fl/fl^ mice) we conclude that IL-10 has a detrimental role, at least in the late phase of POI, as administration of recombinant IL-10 3 h after IM restored the regular POI phenotype.

As our data indicate that IL-10 aggravates POI, we aimed to gain more insight into the mechanism behind this unexpected effect. By the use of the ITIB IL-10 reporter mice (22) we identified infiltrating monocyte-derived macrophages as the major IL-10 producers in the postoperative ME after 24 h while resident muscularis macrophages are the exclusive IL-10 source in the naïve ME. The cellular sources could be confirmed by gene expression studies of flow cytometry sorted cells of WT mice which exhibited the highest expression of IL-10 in monocyte derived macrophages. We further demonstrated that the F4/80^+^Ly6C^+^ cell population predominantly undergoes M2 polarization as it exhibits a strong Arg1 and Ym1 expression after IM and that the M2 marker expression depends on IL-10 which is known to drive M2 polarization also in other inflammatory diseases (36, 37). Notably, IL-4 and IL13 (19, 20, 38, 39), were either not present or much lower upregulated after IM, respectively. Although we did not detect any IL-4 ME transcripts in a time course of up to 72 h after IM, Engel et al. demonstrated a constant IL-4 expression in the ME of naïve and intestinally manipulated mice (26) and Farro et al. observed an IL-4 upregulation but only 12 h after IM (27). This discrepancy may be based on contamination of the ME specimen by submucosal tissue which is known to be continuously express IL-4. However, although we clearly detected IL-4 in the spleen, we could not exclude a higher sensitivity of the quantitative PCR used in the other studies. To ensure that we were not missing any effects of IL-4 on the postoperative M2 polarization, which may be present but technically not detectable, we subjected IL-4^−/−^ mice to IM and observed no difference in ME arginase activity, leukocytes infiltration, and POI outcome compared to WT animals. These data indicate that IL-10 is the major M2 polarization driver during POI. However, our data also indicate that IL-10 affects naïve resident ME macrophages as naïve IL-10^−/−^ mice showed an increased M1 polarization signature. This is line with previous findings showing a higher proinflammatory cytokine expression profile from IL-10 deficient lamina propria macrophages under homeostasis (40). Of note, this M1 signature does not originate from any infiltrating leucocytes as no infiltrating leukocyte were present in the naïve small bowel ME of both IL-10 deficient mouse strains. Only one IL-10^−/−^ mouse spontaneously developed colitis in an age of 10 weeks and was not included in our study. Mice with the LysM-Cre driven leukocyte-restricted IL-10 deficiency were shown not to spontaneously develop an enterocolitis. Therefore, the M1 signature of the naïve ME is highly likely induced by a missing IL-10 ligation on resident macrophages that express higher levels of proinflammatory cytokines.

As we identified monocyte-derived macrophages as the major source but also as an important target of IL-10 during POI, we spend further attention on this cell population. The presence of monocytes in the blood, which are the precursors of the F4/80^+^Ly6C^+^ cells of the postoperative ME, strictly depends on CCR2 (41). Recently two independent publications demonstrated that Ly6C^+^ monocyte ME infiltration during POI is abolished in CCR2^−/−^ mice (27, 42). We hypothesized that an elimination of CCR2 dependent monocytes will also lead to significant loss of IL-10 which may mimic the phenotype of IL-10 deficient mice. In line with the previous studies we also observed an almost complete abrogation of monocyte infiltration by CCR2 deficiency. Confirmingly IL-10 producing Ly6C^+^ monocyte and their Ly6C^+^ F4/80^+^ macrophage descendants were almost completely absent and Arg1 and Ym1 expression were dramatically reduced. However, although the major IL-10 producing cell population was missing in CCR2^−/−^ mice, IL-10 ME protein levels, determined by ELISA whose specificity was confirmed in IL-10^−/−^ mice (Figure 1B), were not reduced but even increased in CCR2^−/−^ mice. Additionally these mice did not reveal any dysmotility 24 h after IM compared to wildtype controls and this phenotype is in line with the findings from Pohl et al. and Farro et al. (27, 42). It should be noted that Farro et al. observed a delayed resolution of POI 72 h after IM in CCR2^−/−^ but we assume that this phenotype is not linked to IL-10 as IL-10 gene expression is not increased in the ME at this time point. Our data suggest that a compensatory source of IL-10 was activated in CCR2^−/−^ mice and resident macrophages appear to be the most likely alternative source as they produce IL-10 and express the IL-10R1 under basal conditions. Of note, the resident macrophages may achieve this compensatory function only in the absence of monocyte-derived IL-10 as their numbers and their IL-10 gene expression rate is not differing in naïve CCR2^−/−^ and WT mice. Another alternative source could be IL-10 producing neutrophils that were increased in the postoperative ME of CCR2^−/−^ mice. Supportingly, neutrophils become more substantially activated in CCR2^−/−^ mice that underwent a Toxoplasma gondii infection and also exhibited reduced monocyte numbers but no reduction in neutrophil numbers (43).

Subsumed, IL-10 drives M2 macrophage polarization but this seems not to be crucial to POI development as an absence of M2 macrophages in CCR2^−/−^ mice did not affect POI. Nevertheless, the question how IL-10 deficiency improves POI remains open. To this end, further insight into the mechanism came from flow cytometry characterization of the leukocyte ME infiltrate. In both transgenic mouse models, the IL-10 deficiency led to a reduction of neutrophils but not monocytes. This phenotype could be reversed by administration of recombinant IL-10 treatment what increased the neutrophil influx in LysM^cre^IL-10^fl/fl^ mice and finally resulted in a normal POI development as it was observed in WT mice. In line with other studies (44, 45), IL-10 alone did not alter neutrophil transmigration *in vitro* indicating that IL-10 does not act as neutrophil chemokine. However, supernatants of traumatized ME specimens of IL-10^−/−^ mice, which were taken into organ culture after *in vivo* manipulation, demonstrated a lower transmigration of WT neutrophils toward surgically manipulated ME supernatant. These findings indicate that IL-10 indirectly affects neutrophil migration and a subsequent comparison of monocytes and neutrophil chemokine expression uncovered a strong reduction of the neutrophil chemokines CXCL1 and CXCL2 but not CCL2, which attracts monocytes. Additionally, inactive neutrophils contain an intracellular pool of the IL-10R that can be mobilized to the cell surface after LPS, TNF-α, or granulocyte/monocyte-colony stimulating factor stimulation (46). Those mediators are also elevated in the postoperative ME (14, 47) and our findings demonstrate a dramatically IM-induced increase in IL-10Rα gene expression in flow-cytometry sorted ME neutrophils but not in monocytes. As the before described neutrophil functions and subcellular localization of the IL10-R can hardly be described *in vivo* we can so far only speculate on this direct function of IL-10 on neutrophils. To this end, IL-10 was shown to impair neutrophil metabolism and bactericidal functions, including phagocytosis and cytotoxicity (48–51). IL-10 was also described to block neutrophil extracellular trap (NET) formation in a model of HIV infection (52). Although the exact mechanism remains to be identified, our data clearly indicate an impairment of neutrophil attraction by an IL-10 mediated reduction of neutrophil chemokine expression.

Our data give new mechanistic insight into an unexpected function of IL-10 in the pathogenesis of POI. However, clinically a direct intervention in the IL-10 pathway may be consequently become a treatment option as IL-10 has also been shown to exert beneficial effects in a variety of diseases and its role in human POI still has to be explored. Nevertheless, IL-10 could be still an interesting target as a potential surrogate marker for POI manifestation or prolongation and it would be interesting to study if IL-10 serum levels remain upregulated or even increase in patients with prolonged POI in comparison to patients without a prolonged POI. Any further speculation on interventional approaches in IL-10 signaling for prevention or treatment of POI are—at this time—rather speculative than evidence-based and additional studies are imperative.

Taken together, the present study demonstrates that IL-10 is a double-edged sword in POI pathophysiology. Basically, IL-10 is mainly produced by resident muscularis macrophages and shapes their polarization. Postoperatively, infiltrating monocyte-derived macrophages emerge as the main IL-10 producers and simultaneously drivers of M2 macrophage polarization. However, while M2 polarization *per se* is not crucial to POI resolution, IL-10 deficiency unexpectedly reduced the postoperative motility disturbances. The underlying mechanisms involve an IL-10-driven indirect alteration of neutrophil migration in POI.

## Author Contributions

KS participated in research design, performed experiments, analyzed results, and prepared the manuscript. ML performed experiments. BS and SS provided IL-10^−/−^ mice and contributed to the manuscript preparation. JK critically revised the manuscript. JH provided the ITIB mice. AR provided the LysMcre/IL10fl/fl mice. TV collected and analyzed the human serum samples. SW contributed to the conception and design of the study, performed experiments, analyzed data, and wrote the manuscript.

### Conflict of Interest Statement

SW and JK received royalties from Wolters Kluwer for contributing and updating articles of the Up2Date clinical resource tool. The remaining authors declare that the research was conducted in the absence of any commercial or financial relationships that could be construed as a potential conflict of interest.

## Abbreviations

Arg: Arginase
GIT: gastrointestinal tract
IL: interleukin
IM: intestinal manipulation
ME: muscularis externa
MPO: myeloperoxidase
POI: postoperative ileus.

## References

[1] Iyer S Saunders WB Stemkowski S. Economic burden of postoperative ileus associated with colectomy in the United States. J Manag Care Pharm. (2009) 15:485–94. 10.18553/jmcp.2009.15.6.485 19610681 PMC10438346

[2] Kalff J Schraut W Simmons R Bauer A. Surgical manipulation of the gut elicits an intestinal muscularis inflammatory response resulting in postsurgical ileus. Ann Surg. (1998) 228:652–63. 10.1097/00000658-199811000-00004 9833803 PMC1191570

[3] van Bree SH Vlug MS Bemelman WA Hollmann MW Ubbink DT Zwinderman AH. Faster recovery of gastrointestinal transit after laparoscopy and fast-track care in patients undergoing colonic surgery. Gastroenterology (2011) 141:872.e1-4–80.e1-4. 21699777 10.1053/j.gastro.2011.05.034

[4] Gomez-Pinilla PJ Farro G Di Giovangiulio M Stakenborg N Nemethova A de Vries A. Mast cells play no role in the pathogenesis of postoperative ileus induced by intestinal manipulation. PLoS ONE (2014) 9:e85304. 10.1371/annotation/99de087d-c3fc-4e9d-9ee9-552b7524f9e1 24416383 PMC3887017

[5] Wehner S Behrendt F Lyutenski B Lysson M Bauer A Hirner A. Inhibition of macrophage function prevents intestinal inflammation and postoperative ileus in rodents. Gut (2007) 56:176–85. 10.1136/gut.2005.089615 16809419 PMC1856749

[6] Stoffels B Hupa KJ Snoek SA van Bree S Stein K Schwandt T. Postoperative ileus involves interleukin-1 receptor signaling in enteric glia. Gastroenterology (2014) 146:176.e1–87.e1. 10.1053/j.gastro.2013.09.030 24067878

[7] Boeckxstaens GE de Jonge WJ. Neuroimmune mechanisms in postoperative ileus. Gut (2009) 58:1300–11. 10.1136/gut.2008.169250 19671558

[8] Wehner S Vilz TO Stoffels B Kalff JC. Immune mediators of postoperative ileus. Langenbecks Arch Surg. (2012) 397:591–601. 10.1007/s00423-012-0915-y 22382699

[9] Henderson RB Hobbs JA Mathies M Hogg N. Rapid recruitment of inflammatory monocytes is independent of neutrophil migration. Blood (2003) 102:328–35. 10.1182/blood-2002-10-3228 12623845

[10] Ingersoll MA Platt AM Potteaux S Randolph GJ. Monocyte trafficking in acute and chronic inflammation. Trends Immunol. (2011) 32:470–7. 10.1016/j.it.2011.05.001 21664185 PMC3179572

[11] Kolaczkowska E Kubes P. Neutrophil recruitment and function in health and inflammation. Nat Rev Immunol. (2013) 13:159–75. 10.1038/nri3399 23435331

[12] van Bree SH Nemethova A Cailotto C Gomez-Pinilla PJ Matteoli G Boeckxstaens GE. New therapeutic strategies for postoperative ileus. Nat Rev Gastroenterol Hepatol. (2012) 9:675–83. 10.1038/nrgastro.2012.134 22801725

[13] Stein K Stoffels M Lysson M Schneiker B Dewald O Kronke G. A role for 12/15-lipoxygenase-derived proresolving mediators in postoperative ileus: protectin DX-regulated neutrophil extravasation. J Leukoc Biol. (2016) 99:231–9. 10.1189/jlb.3HI0515-189R 26292977

[14] Stoffels B Schmidt J Nakao A Nazir A Chanthaphavong R Bauer A. Role of interleukin 10 in murine postoperative ileus. Gut (2009) 58:648–60. 10.1136/gut.2008.153288 19359433

[15] Mills CD Kincaid K Alt JM Heilman MJ Hill AM. M-1/M-2 macrophages and the Th1/Th2 paradigm. J Immunol. (2000) 164:6166–73. 10.4049/jimmunol.164.12.6166 10843666

[16] Mills CD. M1 and M2 macrophages: oracles of health and disease. Crit Rev Immunol. (2012) 32:463–88. 10.1615/CritRevImmunol.v32.i6.10 23428224

[17] Mantovani A Garlanda C Locati M. Macrophage diversity and polarization in atherosclerosis a question of balance. Arterioscler Thromb Vasc Biol. (2009). 29:1419–23. 10.1161/ATVBAHA.108.180497 19696407

[18] Sica A Mantovani A. Macrophage plasticity and polarization: *in vivo* veritas. J Clin Invest. (2012) 122:787–95. 10.1172/JCI59643 22378047 PMC3287223

[19] Gordon S. Alternative activation of macrophages. Nat Rev Immunol. (2003) 3:23–35. 10.1038/nri978 12511873

[20] Mantovani A Sica A Sozzani S Allavena P Vecchi A Locati M. The chemokine system in diverse forms of macrophage activation and polarization. Trends Immunol. (2004) 25:677–86. 10.1016/j.it.2004.09.015 15530839

[21] Gordon S Martinez FO. Alternative activation of macrophages: mechanism and functions. Immunity (2010) 32:593–604. 10.1016/j.immuni.2010.05.007 20510870

[22] Bouabe H Liu Y Moser M Bösl M Heesemann J. Novel highly sensitive IL-10-beta-lactamase reporter mouse reveals cells of the innate immune system as a substantial source of IL-10 *in vivo*. J Immunol. (2011) 187:3165–76. 10.4049/jimmunol.1101477 21844394

[23] Corraliza I Campo M Soler G Modolell M. Determination of arginase activity in macrophages: a micromethod. J Immunol Methods (1994) 174:231–5. 10.1016/0022-1759(94)90027-2 8083527

[24] Swamydas M Lionakis MS Isolation, purification and labeling of mouse bone marrow neutrophils for functional studies and adoptive transfer experiments. J Vis Exp. (2013) 10:e50586 10.3791/50586 PMC3732092 23892876

[25] Clausen BE Burkhardt C Reith W Renkawitz R Forster I. Conditional gene targeting in macrophages and granulocytes using LysMcre mice. Transgenic Res. (1999) 8:265–77. 10.1023/A:1008942828960 10621974

[26] Engel D Koscielny A Wehner S Maurer J Schiwon M Franken L. T helper type 1 memory cells disseminate postoperative ileus over the entire intestinal tract. Nat Med. (2010) 16:1407–13. 10.1038/nm.2255 21113155

[27] Farro G Stakenborg M Gomez-Pinilla PJ Labeeuw E Goverse G Di Giovangiulio M. CCR2-dependent monocyte-derived macrophages resolve inflammation and restore gut motility in postoperative ileus. Gut (2017) 66:2098–109. 10.1136/gutjnl-2016-313144 28615302

[28] Kato M Honda I Suzuki H Murakami M Matsukawa S Hashimoto Y. Interleukin-10 production during and after upper abdominal surgery. J Clin Anesth. (1998) 10:184–8. 10.1016/S0952-8180(97)00264-X 9603586

[29] Vilz TO Roessel L Chang J Pantelis D Schwandt T Koscielny A. Establishing a biomarker for postoperative ileus in humans - results of the BiPOI trial. Life Sci. (2015) 143:58–64. 10.1016/j.lfs.2015.10.024 26596561

[30] Eming SA Werner S Bugnon P Wickenhauser C Siewe L Utermohlen O. Accelerated wound closure in mice deficient for interleukin-10. Am J Pathol. (2007) 170:188–202. 10.2353/ajpath.2007.060370 17200193 PMC1762712

[31] Cross M Mangelsdorf I Wedel A Renkawitz R. Mouse lysozyme M gene: isolation, characterization, and expression studies. Proc Natl Acad Sci USA. (1988) 85:6232–6. 10.1073/pnas.85.17.6232 3413093 PMC281943

[32] Malvin NP Seno H Stappenbeck TS. Colonic epithelial response to injury requires Myd88 signaling in myeloid cells. Mucosal Immunol. (2012) 5:194–206. 10.1038/mi.2011.65 22258450 PMC3791628

[33] Lu N Wang L Cao H Liu L van Kaer L Washington MK. Activation of the epidermal growth factor receptor in macrophages regulates cytokine production and experimental colitis. J Immunol. (2014) 192:1013–23. 10.4049/jimmunol.1300133 24391216 PMC4006992

[34] Walley KR Lukacs NW Standiford TJ Strieter RM Kunkel SL. Balance of inflammatory cytokines related to severity and mortality of murine sepsis. Infect Immun. (1996) 64:4733–8. 8890233 10.1128/iai.64.11.4733-4738.1996 PMC174439

[35] Steinhauser ML Hogaboam CM Kunkel SL Lukacs NW Strieter RM Standiford TJ. IL-10 is a major mediator of sepsis-induced impairment in lung antibacterial host defense. J Immunol. (1999) 162:392–9. 9886412

[36] Fujisaka S Usui I Bukhari A Ikutani M Oya T Kanatani Y. Regulatory mechanisms for adipose tissue M1 and M2 macrophages in diet-induced obese mice. Diabetes (2009) 58:2574–82. 10.2337/db08-1475 19690061 PMC2768159

[37] Yang Y Wang X Huycke T Moore DR Lightfoot SA Huycke MM. Colon macrophages polarized by commensal bacteria cause colitis and cancer through the bystander effect. Transl Oncol. (2013) 6:596–606. 10.1593/tlo.13412 24151540 PMC3799201

[38] van der Poll T de Waal Malefyt R Coyle S Lowry S. Antiinflammatory cytokine responses during clinical sepsis and experimental endotoxemia: sequential measurements of plasma soluble interleukin (IL)-1 receptor type II, IL-10, and IL-13. J Infect Dis. (1997) 175:118–22. 10.1093/infdis/175.1.118 8985204

[39] Martinez F Helming L Gordon S. Alternative activation of macrophages: an immunologic functional perspective. Ann Rev Immunol. (2009) 27:451–83. 10.1146/annurev.immunol.021908.132532 19105661

[40] Zigmond E Bernshtein B Friedlander G Walker CR Yona S Kim KW Macrophage-restricted interleukin-10 receptor deficiency, but not IL-10 deficiency, causes severe spontaneous colitis. Immunity (2014) 40:720–33. 10.1016/j.immuni.2014.03.012 24792913

[41] Serbina NV Pamer EG. Monocyte emigration from bone marrow during bacterial infection requires signals mediated by chemokine receptor CCR2. Nat Immunol. (2006) 7:311–7. 10.1038/ni1309 16462739

[42] Pohl JM Gutweiler S Thiebes S Volke JK Klein-Hitpass L Zwanziger D. Irf4-dependent CD103(+)CD11b(+) dendritic cells and the intestinal microbiome regulate monocyte and macrophage activation and intestinal peristalsis in postoperative ileus. Gut (2017) 66:2110–20. 10.1136/gutjnl-2017-313856 28615301 PMC5749346

[43] Grainger JR Wohlfert EA Fuss IJ Bouladoux N Askenase MH Legrand F. Inflammatory monocytes regulate pathologic responses to commensals during acute gastrointestinal infection. Nat Med. (2013) 19:713–21. 10.1038/nm.3189 23708291 PMC3755478

[44] Crepaldi L Gasperini S Lapinet JA Calzetti F Pinardi C Liu Y. Up-regulation of IL-10R1 expression is required to render human neutrophils fully responsive to IL-10. J Immunol. (2001) 167:2312–22. 10.4049/jimmunol.167.4.2312 11490020

[45] Cassatella MA Tamassia N Crepaldi L McDonald PP Ear T Calzetti F. Lipopolysaccharide primes neutrophils for a rapid response to IL-10. Eur J Immunol. (2005) 35:1877–85. 10.1002/eji.200526088 15864776

[46] Elbim C Reglier H Fay M Delarche C Andrieu V El Benna J. Intracellular pool of IL-10 receptors in specific granules of human neutrophils: differential mobilization by proinflammatory mediators. J Immunol. (2001) 166:5201–7. 11290804 10.4049/jimmunol.166.8.5201

[47] Kalff JC Turler A Schwarz NT Schraut WH Lee KK Tweardy DJ. Intra-abdominal activation of a local inflammatory response within the human muscularis externa during laparotomy. Ann Surg. (2003) 237:301–15. 10.1097/01.SLA.0000055742.79045.7E 12616113 PMC1514322

[48] Kasama T Strieter R Lukacs N Burdick M Kunkel S. Regulation of neutrophil-derived chemokine expression by IL-10. J Immunol. (1994) 152:3559–69. 8144935

[49] Laichalk L Danforth J Standiford T. Interleukin-10 inhibits neutrophil phagocytic and bactericidal activity. FEMS Immunol Med Microbiol. (1996) 15:181–7. 10.1111/j.1574-695X.1996.tb00084.x 8908479

[50] Capsoni F Minonzio F Ongari A Carbonelli V Galli A Zanussi C. Interleukin-10 down-regulates oxidative metabolism and antibody-dependent cellular cytotoxicity of human neutrophils. Scand J Immunol. (1997) 45:269–75. 10.1046/j.1365-3083.1997.d01-393.x 9122616

[51] Cassatella M. The neutrophil: one of the cellular targets of interleukin-10. Int J Clin Lab Res. (1998) 28:148–61. 10.1007/s005990050036 9801925

[52] Saitoh T Komano J Saitoh Y Misawa T Takahama M Kozaki T. Neutrophil extracellular traps mediate a host defense response to human immunodeficiency virus-1. Cell Host Microbe (2012) 12:109–16. 10.1016/j.chom.2012.05.015 22817992

