# Supplementary material for: Leukocyte-Derived Interleukin-10 Aggravates Postoperative Ileus
Source: Front Immunol. 2018 Nov 13;9:2599. doi: 10.3389/fimmu.2018.02599 (PMC6294129; doi:10.3389/fimmu.2018.02599)
Supplement: Supplementary file 1 [file Data_Sheet_1.PDF]

Supplemental Figure 1

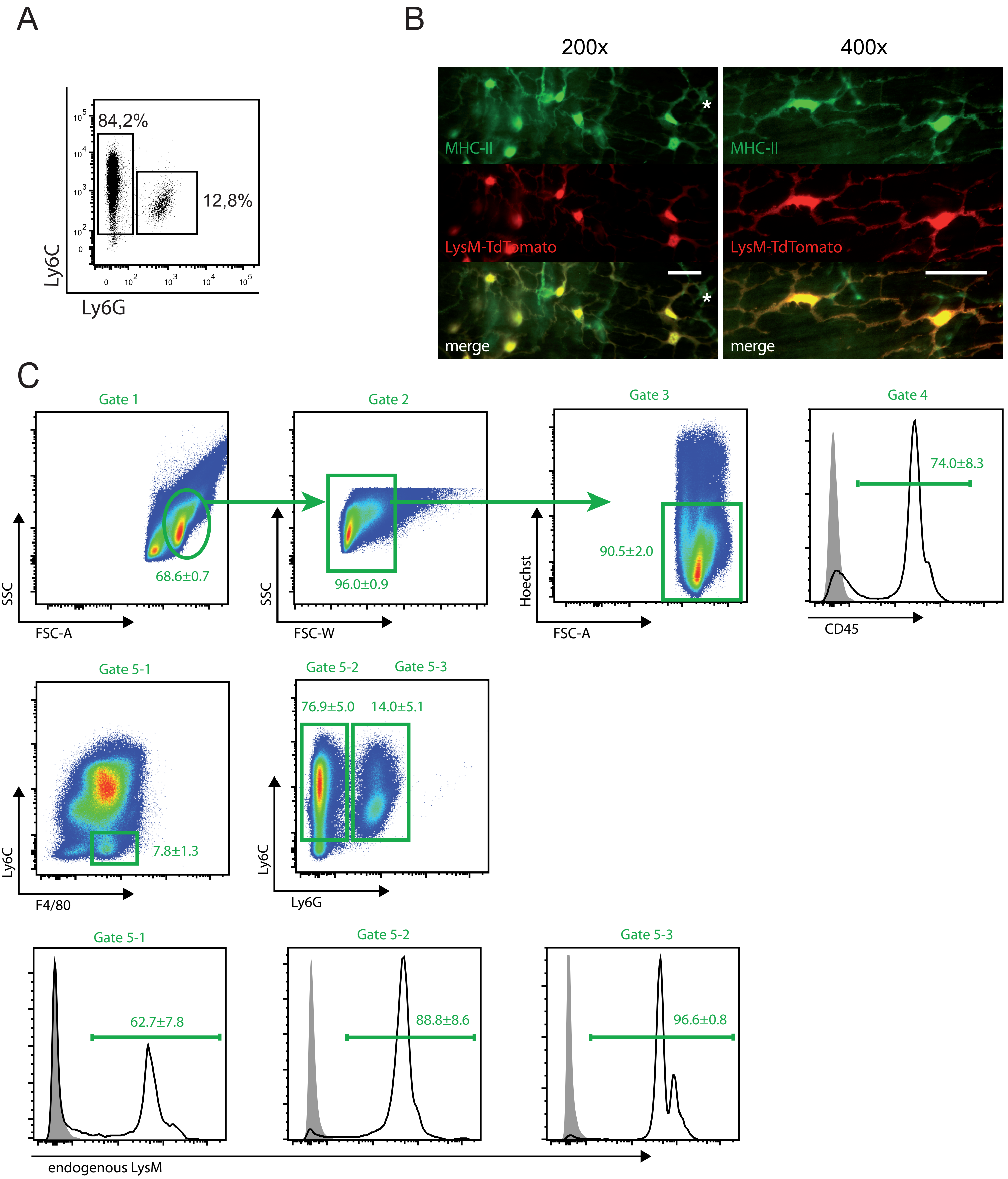

Supplemental Figure 1: (A) Relative quantification of leukocyte populations within the intestinally manipulated (IM24h) ME of WT mice. (B) LysM is expressed by leukocytes of the non-manipulated and intestinal manipulated ME. Representative immunofluorescence staining for MHCII and endogenous LysM in LysMcre-TdTomatoSTOPfl/fl mice within an ME whole mount specimen of untreated WT mice. (C) Flow cytometry analysis of endogenous ysM expression in F4/80+ (5-1), Ly6C+Ly6G- (5-2) and Ly6C+Ly6G+ (5-3) Hoechst- CD45+ ME leukocytes in LysMcre-TdTomatoSTOPfl/fl mice 24h after IM. Plots are representative for two independent mice, whereby gates indicate means ± SD (% of living CD45+ cells).
