# Supplementary material for: Leukocyte-Derived Interleukin-10 Aggravates Postoperative Ileus
Source: Front Immunol. 2018 Nov 13;9:2599. doi: 10.3389/fimmu.2018.02599 (PMC6294129; doi:10.3389/fimmu.2018.02599)
Supplement: Supplementary file 2 [file Data_Sheet_2.PDF]

Supplemental Figure 2

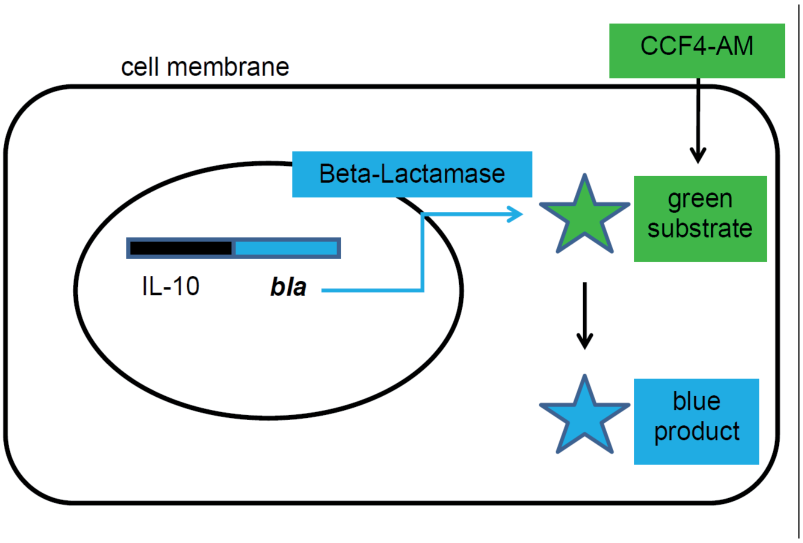

Supplemental Figure 2: Scheme of ITIB+/- reporter mice indicating IL-10 activity. Beta-lactamase substrate CCF4-AM is a fluorescence energy transfer (FRET) dye that results in green emission at 520nm when given to living ME cells. During presence of beta-lactamase activity that is limited to IL-10 expressing cells, FRET is disrupted and CCF4-AM emission switches to 450nm blue fluorescence signal.
