# Supplementary material for: Leukocyte-Derived Interleukin-10 Aggravates Postoperative Ileus
Source: Front Immunol. 2018 Nov 13;9:2599. doi: 10.3389/fimmu.2018.02599 (PMC6294129; doi:10.3389/fimmu.2018.02599)
Supplement: Supplementary file 3 [file Data_Sheet_3.PDF]

# Supplemental Figure 3

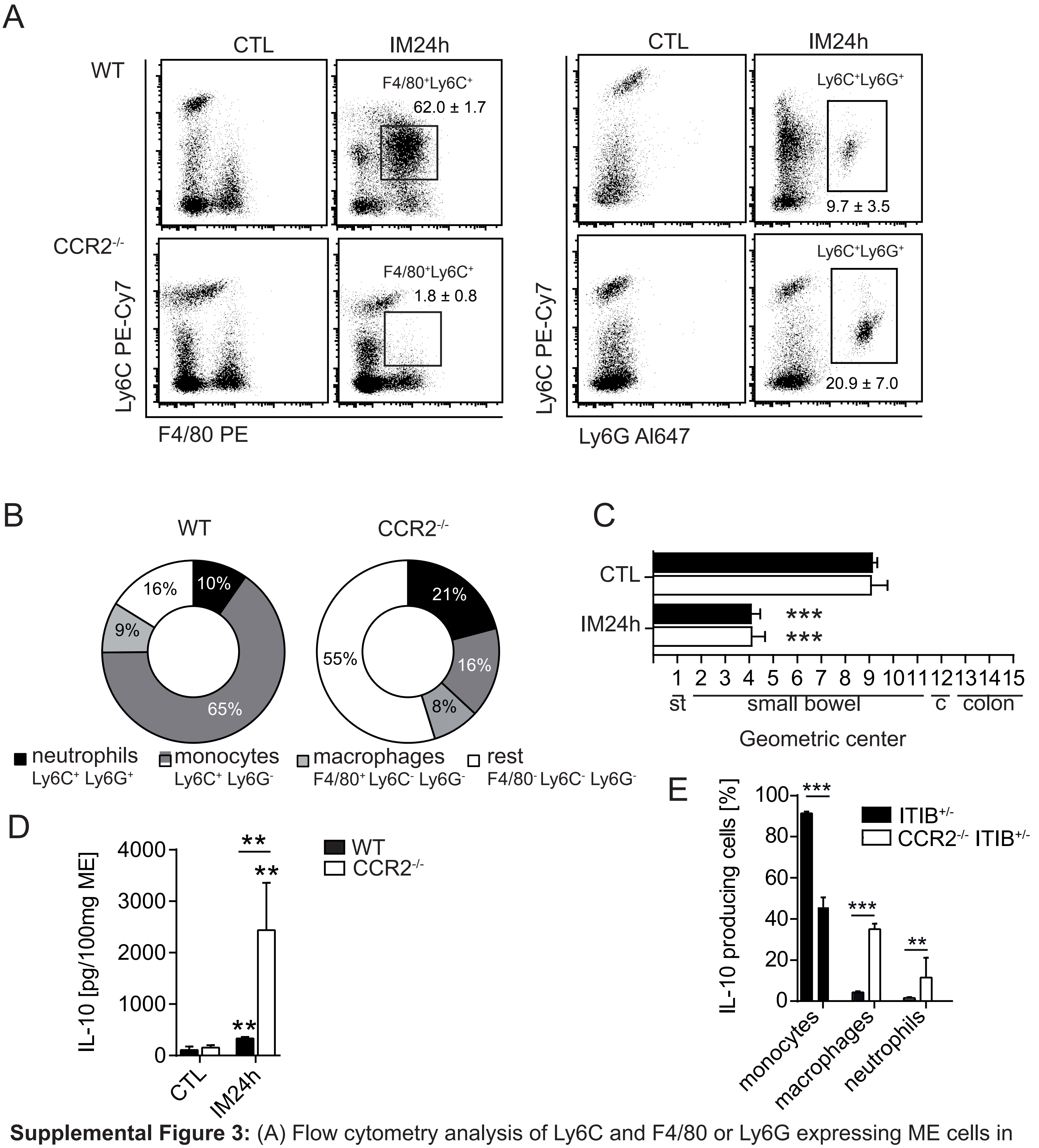


**Supplemental Figure 3:** (A) Flow cytometry analysis of Ly6C and F4/80 or Ly6G expressing ME cells in WT and CCR2-/- mice 24h after IM or in naïve controls (CTL). Plots are representative for five mice, whereby gates indicate means ± SD (% of living cells). (B) Relative quantification of leukocyte populations within the intestinally manipulated (IM24h) ME of CCR2-/- compared to WT mice. (C) GI-transit was quantified 24h postoperatively in comparison to unmanipulated animals (CTL) and plotted as mean ± SEM calculated by the geometric centers of distribution of a fluid meal. st = stomach, c = cecum. n=5 for all groups. Statistical analysis was performed by one-way ANOVA followed by Bonferroni's post-hoc test (***$p<0.001$ vs. CTL). (D) IL-10 release into the media of 24h organ cultured ME harvested from intestinal manipulated (IM24h) WT or CCR2-/- mice. Groups (n=3-4) were compared via unpaired t-test (**$p<0.01$). Bar graphs demonstrate means ± SEM. (E) Percentage of IL-10 producing cells in the ME of CCR-2 deficient or CCR-2 competent ITIB+/- mice 24h after IM. n=5 for for all groups. Samples were analyzed by Student's t test or one-way analysis of variance including Bonferroni post-hoc test
