# Supplementary material for: Leukocyte-Derived Interleukin-10 Aggravates Postoperative Ileus
Source: Front Immunol. 2018 Nov 13;9:2599. doi: 10.3389/fimmu.2018.02599 (PMC6294129; doi:10.3389/fimmu.2018.02599)
Supplement: Supplementary file 4 [file Data_Sheet_4.PDF]

Supplemental Figure 4

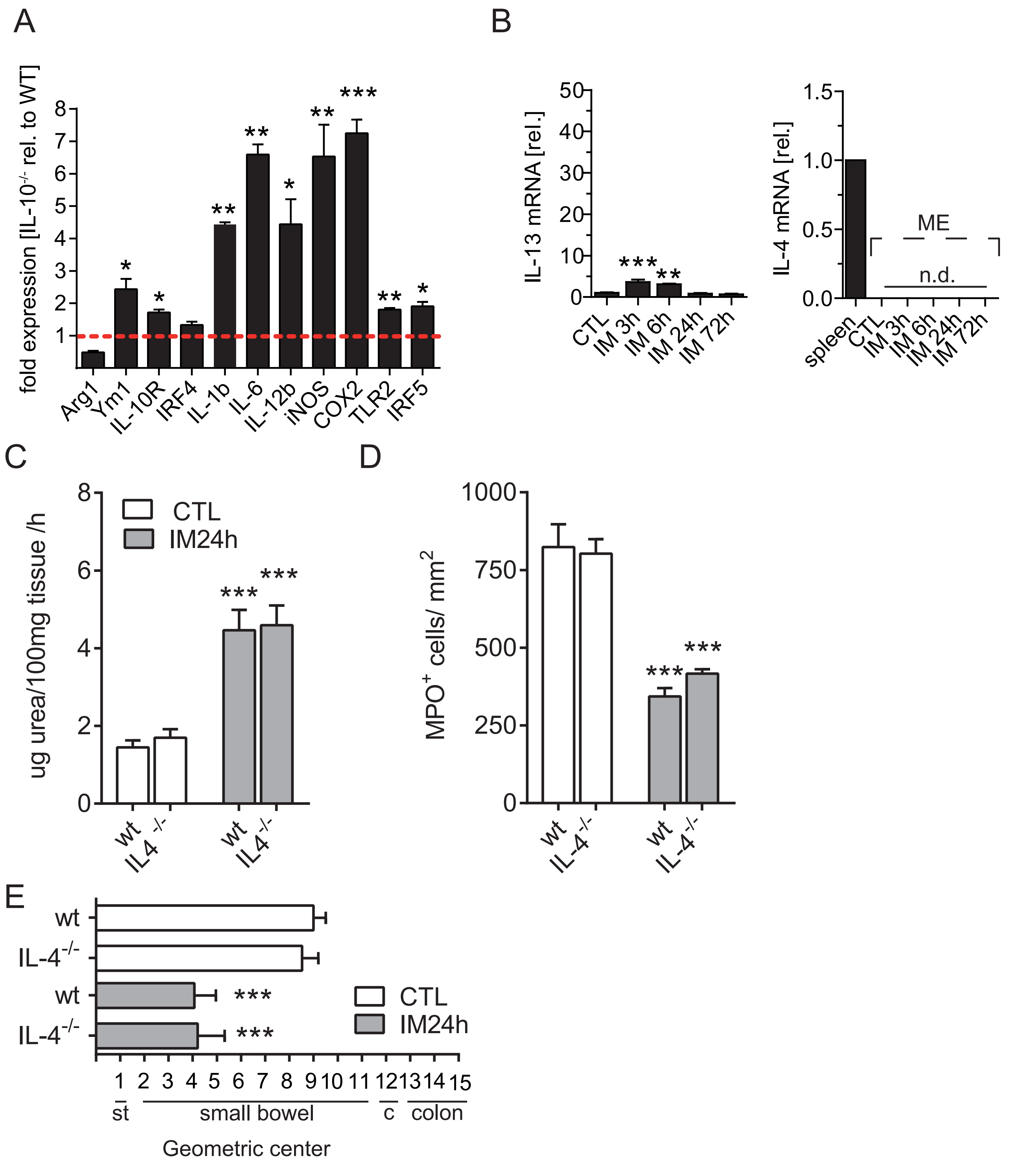

Supplemental Figure 4: (A) Resident F4/80+Ly6C- macrophages were sorted by flow cytometry from naïve WT and IL-10-/- animals. Indicated cell populations underwent gene expression analysis of Arg1, Ym1, IL-10Rα, IRF4, IL-1β, IL-6, IL-12β, iNOS, COX2, TLR2 and IRF5. n=3 for all groups. Statistical analysis was done by unpaired t-test. *p<0.05, **p<0.01 and ***p<0.001 vs. WT mice. (B) Gene expression of IL-13 and IL-4 was analyzed in WT mice after indicated time points and compared to naïve controls (CTL) or spleen, respectively. n=5 mice per group. Statistical analysis was done by one-way ANOVA followed by Bonferroni's post-hoc test. **p<0.01 and ***p<0.001; n.d. not detected vs. controls (CTL). (C) Arginase activity was determined by urea formation within naïve and manipulated WT and IL-4-/- ME specimens. (D) Quantification of MPO+ cells within the same animals used in (C). (E) GI-transit was plotted as mean ± SEM calculated by the geometric centers of distribution of a fluorescent marker. st = stomach, c = cecum. Bar graphs show means ± SEM and are representative for five independent experiments. Statistical analysis was performed with a one-way ANOVA followed by Bonferroni's post-hoc test (***p<0.001 vs. CTL or indicated groups).
