# Supplementary material for: Leukocyte-Derived Interleukin-10 Aggravates Postoperative Ileus
Source: Front Immunol. 2018 Nov 13;9:2599. doi: 10.3389/fimmu.2018.02599 (PMC6294129; doi:10.3389/fimmu.2018.02599)
Supplement: Supplementary file 5 [file Table_1.docx]

**Supplementary table 1**

Primer sequences and Taqman probes used for quantitative RT-PCR

| **Gene name** | **sense** | **antisense** |
| --- | --- | --- |
| IL-10 | 5’-GATGCCCCAGGCAGAGAA-3’ | 5’-CACCCAGGGAATTCAAATGC-3’ |
| CXCL1 | 5’-TCTCCGTTACTTGGGGACAC-3’ | 5’-CCACACTCAAGAATGGTCGC-3’ |
| CXCL2 | 5’-TCCAGGTCAGTTAGCCTTGC-3’ | 5’- CGGTCAAAAAGTTTGCCTTG-3’ |
| IRF4 | 5’-CAAAGCACAGAGTCACCTGG-3’ | 5’-TGCAAGCTCTTTGACACACA-3’ |
| IRF5 | 5’-CAGGTTGGCCTTCCACTTG-3’ | 5’-ATGGGGACAACACCATCTTC-3’ |
| IL-12b | 5'-CCTGAAGTGTGAAGCACCAA-3' | 5'-TCAGGGGAACTGCTACTGCT-3' |
| TLR2 | 5`-CTGCTTTCCTGCTGGAGATTT-3` | 5`-TGTAACGCAACAGCTTCAGG-3` |

| **Gene name** | **Quantitect Primer Assay ID** |
| --- | --- |
| Arg-1 | QT00134288 |
| IL-4 | QT02241722 |
| IL-6 | QT00098875 |
| IL-13 | QT02423449 |
| IL-10Rα | QT00112742 |
|  | Tagman probe assay |

| **Gene name** | **Tagman Probes Assay ID** |
| --- | --- |
| IL-1β | Mm00434228_m1 |
| CCL-2 | Mm00441242_m1 |
| COX2 | Mm00478374_m1 |
| iNOS | Mm00440485_m1 |
| GAPDH | Mm99999915_g1 |
