# Supplementary material for: Leukocyte-Derived Interleukin-10 Aggravates Postoperative Ileus
Source: Front Immunol. 2018 Nov 13;9:2599. doi: 10.3389/fimmu.2018.02599 (PMC6294129; doi:10.3389/fimmu.2018.02599)
Supplement: Supplementary file 6 [file Table_2.docx]

**Supplementary table 2**

List of antibodies used in this study.

| **Protein name** | **Dye/Diluation** | **Use** | **Clone and Company** |
| --- | --- | --- | --- |
| MHC-II | no dye / 1:100 | IHC | M5/114.15.2, Biolegend |
| F4/80 | no dye / 1:200 | IHC | BM8, Life Technolgies |
| Arg-1 | no dye / 1:100 | IHC | N-20, Santa Cruz Biotechnology |
| CD45 | PerCP/Cy5.5 / 1:200 | FC | 30-F11m eBiosciences |
| F4/80 | FITC or PE / 1:200 | FC | BM8, Biolegend |
| Ly6C | PE-Cy7 / 1:200 | FC | HK1.4, Biolegend |
| Ly6G | Alexa647 / 1:300 | FC | 1A8, Biolegend |

IHC: Immunohistochemistry, FC: flow cytometry
